# Lean MAFLD as an independent risk factor for carotid plaque: The value of FIB-4 index in subgroup analysis

**DOI:** 10.1371/journal.pone.0343781

**Published:** 2026-11-05

**Authors:** Guanqun Chao, Ailing Lin, Tianxiang Mai

**Affiliations:** 1 Department of General Practice, Zhejiang University School of Medicine Sir Run Run Shaw Hospital, Hangzhou, China; 2 Department of International Outpatient, Zhejiang University School of Medicine Sir Run Run Shaw Hospital, Hangzhou, China; Kaohsiung Medical University Hospital, TAIWAN

## Abstract

**Objective:**

This study aimed to investigate the associations between different subtypes of Metabolic dysfunction-associated fatty liver disease (MAFLD) and carotid plaque, and to evaluate the independent association of the Fibrosis-4 (FIB-4) index with carotid plaque risk.

**Methods:**

Multivariable logistic regression was used to assess associations of MAFLD subtypes and the FIB-4 index with carotid plaque. Two covariate‑selection strategies were adopted: confounders for MAFLD‑subtype analyses were chosen based on clinical considerations, while subgroup‑analysis confounders were screened using a univariable p < 0.10 threshold. Separate adjusted models were constructed disentangle the independent association of the composite FIB-4 score from its individual components.

**Results:**

A total of 15,544 participants were included. After multivariable adjustment, lean MAFLD demonstrated an independent association with carotid plaque (OR=1.444), and its point estimate was numerically higher than those for diabetes-related MAFLD (OR = 1.354) and overweight/obesity-related MAFLD (OR = 1.172), although the confidence intervals overlapped. The FIB-4 index demonstrated a significant association with carotid plaque across all MAFLD subtypes (all p < 0.05) in models excluding its individual constituent components (OR range: 1.401–2.227).

**Conclusion:**

Lean MAFLD is independently associated with carotid plaque. The FIB-4 index, a readily accessible noninvasive marker, shows consistent associations with carotid plaque across all MAFLD subtypes.

## 1 Introduction

Metabolic dysfunction-associated fatty liver disease (MAFLD) was first proposed in 2020, followed by metabolic-associated steatotic liver disease (MASLD), the updated diagnostic framework released in 2023; both systems were designed to revise the traditional definition of non-alcoholic fatty liver disease [1]. Although both MAFLD and MASLD were developed to revise the traditional diagnostic framework of non-alcoholic fatty liver disease (NAFLD), the MAFLD system was constructed with a focus on screening subjects with obvious metabolic dysfunction and covers a wide range of patient subgroups [2]. Several cohort studies have reported a higher proportion of lean individuals among patients meeting MASLD criteria relative to those diagnosed under MAFLD standards [3]. In a recent longitudinal study, it was found that MAFLD increases the overall risk of death rather than MASLD, with metabolic components being the main factors increasing the risk of death, while cancer and cardiovascular disease are common causes of death [4]. Patients diagnosed under either MAFLD or MASLD criteria are prone to both hepatic lesions and extrahepatic complications, and this spectrum of manifestations imposes a substantial burden on public health systems.

Carotid atherosclerotic features are closely correlated with the severity of coronary hemodynamic abnormalities, which makes carotid plaque evaluation a valuable marker to identify patients at high risk of coronary artery disease [5]. Previous studies have suggested that non-alcoholic fatty liver disease and MAFLD are associated with atherosclerosis [6]. All participants in this cohort were enrolled following the MAFLD diagnostic standards adopted at data collection; thus MAFLD is consistently applied throughout the manuscript to match our inclusion criteria. Recent recommendations on MASLD management suggest that Fibrosis-4 (FIB-4) can be used as the preferred indicator for fibrotic stratification [7]. A significant positive association was observed between the FIB-4 index and carotid atherosclerosis in patients with diabetes mellitus [8]. Accumulating evidence indicates that the FIB-4 score, a surrogate marker of liver fibrosis, is independently correlated with subclinical carotid atherosclerosis among subjects with no prior diagnosis of hepatic disorders [9].This study aims to further investigate the close association between MAFLD subgroup and carotid plaque by analyzing the association between MAFLD and carotid plaque, and the important value of FIB-4 for carotid plaque.

## 2 Materials and methods

### 2.1 Sample data source

The data included in this study were from the population who underwent physical examinations at the Health Promotion Center of our hospital from 01/01/2019–31/07/2023. Data access for research purposes occurred on 17/05/2024. This retrospective study was approved by Sir Run Run Shaw Hospital Ethics Committee (Approval No. 2024 Yan No. 0296). All participants received verbal information about physical examination procedures at the time of their routine health check‑up. All personal identifying information was de‑identified prior to data analysis.

### 2.2 Study population classification

Diagnostic criteria for metabolic dysfunction‑associated fatty liver disease (MAFLD) were adopted from an internationally endorsed expert consensus published in 2020 [10]. Identification of hepatic fat accumulation via abdominal ultrasound constitutes the essential precondition for assigning an MAFLD diagnosis. An individual qualifies for MAFLD when hepatic steatosis is confirmed alongside at least one of three clinical scenarios: having overweight or obesity, a confirmed type 2 diabetes mellitus (T2DM) diagnosis, or detectable metabolic dysfunction.

Metabolic dysfunction is defined as the presence of two or more out of seven predefined cardiometabolic risk markers. Notably, this requirement for ≥2 metabolic risk abnormalities (only applies to participants who are neither overweight/obese nor living with T2DM). Individuals with overweight/obesity or T2DM can fulfil MAFLD diagnostic criteria based on hepatic steatosis alone, without additional metabolic risk‑factor requirements. The seven risk markers cover anthropometric, blood‑pressure, lipid, glucose‑related, inflammatory and insulin‑resistance parameters, as listed below:

1) Elevated waist circumference (cut‑offs ≥90 cm for Asian men and ≥80 cm for Asian women);
2) Blood‑pressure readings reaching or exceeding 130/85 mmHg, or ongoing antihypertensive pharmacological intervention;
3) Fasting triglyceride levels ≥1.70 mmol/L or receipt of lipid‑lowering agents;
4) Reduced high‑density‑lipoprotein cholesterol concentrations: below 1.03 mmol/L in male individuals and below 1.29 mmol/L in female individuals, or use of lipid‑modifying medication;
5) Laboratory findings consistent with prediabetic glycaemic status;
6) Evidence of insulin resistance reflected by a HOMA‑IR value of 2.5 or higher;
7) Raised high‑sensitivity C‑reactive protein (hs‑CRP) exceeding 2 mg/L.

Since fasting‑insulin assays were not included within the routine physical‑examination test battery for this retrospective cohort, calculation of the HOMA‑IR index could not be accomplished, and this marker was therefore excluded from our metabolic risk assessment. For participants without overweight/obesity and without T2DM, metabolic dysfunction status was assessed using the remaining six markers available in our dataset.

Subgroup analyses were restricted exclusively to participants who had fulfilled all requirements for an MAFLD diagnosis. The MAFLD group was further stratified into the following three subgroups:

MAFLD with type 2 diabetes (T2DM): Participants meeting the diagnostic criteria for both MAFLD and T2DM.

MAFLD with overweight/obesity: Participants with a body mass index (BMI) ≥ 23 kg/m^2^ who fulfilled the MAFLD criteria but did not have T2DM.

Lean MAFLD: Participants diagnosed with MAFLD who had a BMI < 23 kg/m^2^ and no T2DM.

The BMI cutoff of 23 kg/m^2^ for lean fatty liver was adopted based on diagnostic standards for Asian populations, which differ from the thresholds applied to Western populations.

Ultrasound‑based diagnostic assessment: Liver ultrasound and carotid artery sonography were performed by on‑site certified sonographers as part of routine health‑check examinations in our hospital. For this retrospective database‑based study, we adopted the final clinical diagnostic conclusions reported in the original ultrasound medical records. Participants with a clinical diagnosis of hepatic steatosis on liver ultrasound were regarded as having hepatic fat accumulation for MAFLD diagnosis. Participants with a reported diagnosis of carotid plaque in carotid sonography records were defined as the carotid‑plaque group, while those without a plaque diagnosis constituted the non‑carotid‑plaque control group.

### 2.3 Inclusion and exclusion criteria

Participants were included if they met all of the following criteria:

(1) Age ≥ 18 years;
(2) Underwent a standardized physical examination and medical history review by a qualified general practitioner;
(3) Completed a hepatic ultrasound examination;
(4) Underwent laboratory blood tests.

Participants were excluded based on any of the following:

(1) Missing baseline demographic data (e.g., age or gender);
(2) Incomplete medical history records;
(3) Insufficient physical examination data;
(4) Incomplete laboratory test results;
(5) Absence of carotid ultrasound examination;
(6) Use of immunosuppressants, antineoplastic agents, or other excluded medications within the past 3 months;
(7) Diagnosis of any of the following conditions: malignant tumors, connective tissue diseases, severe infection, etc.
(8) Participants with clearly documented heavy alcohol intake (male ≥30 g pure ethanol per day, female ≥20 g pure ethanol per day) were excluded from analysis.

### 2.4 Categories of data included

(1) Basic information: gender, age, disease history, surgical history, medication history, smoking history, alcohol drinking history; (2) Blood tests: platelets, liver function, blood glucose, glycated hemoglobin, blood lipids, 25(OH)D, uric acid; (3) Ultrasound: liver ultrasound, carotid artery ultrasound; (4) Physical examination: height, weight, waist circumference, blood pressure.

Smoking and alcohol consumption were extracted from self-reported medical questionnaires and recorded as binary variables.

### 2.5 Indicators of special categories

Body mass index (BMI) was calculated as weight in kilograms divided by height in meters squared:

BMI=Weight(kg)/[Height(m)]^2^

The Fibrosis-4 (FIB-4) index was calculated using the following formula [7]:

FIB-4 =(Age[years] × AST[U/L])/(**Platelet count** [10^9^/L]×(ALT [U/L])^1/2^）

### 2.6 Statistical analyses

Statistical analyses were conducted following a stepwise approach. Normality of continuous variables was assessed using Shapiro-Wilk tests, with parametric (Student’s t-test) and non-parametric (Mann-Whitney U test) methods applied accordingly. Categorical variables were compared using χ^2^ or Fisher’s exact tests. Two different variable-selection strategies for multivariable logistic regression were applied according to distinct research objectives.

For analyses evaluating the association between MAFLD subtypes and carotid‑plaque risk, covariates were pre‑specified based on the study hypothesis. Non‑modifiable demographic factors (age, gender) and modifiable lifestyle factors (smoking status, alcohol consumption) were included as core confounders, regardless of univariable p‑values.

For the subgroup multivariable models presented in Tables 3–5 (exploring the relationship between FIB-4 and carotid plaque within each MAFLD subtype), candidate covariates were screened via univariable analysis. Variables with p < 0.1 in univariable testing were retained as candidate confounders for multivariable regression to avoid omitting variables with potential clinical relevance. To evaluate the independent association of the composite FIB-4 index with carotid‑plaque risk, two multivariable logistic regression models were fitted for each MAFLD subgroup using an identical pool of candidate confounders derived from the univariable p < 0.10 screening. In Model 1, candidate confounders were included without the composite FIB-4 index. In Model 2, the composite FIB-4 index was added to the same set of candidate confounders. Any of the four constituent parameters of FIB-4 (age, platelet count, AST, ALT) that had been selected during univariable‑screening were removed from Model 2, to mitigate multicollinearity, since all four variables enter into the calculation of the composite FIB-4 index.Notably, chronological age is embedded within the FIB-4 formula and is not included as a separate covariate in Model 2; therefore, reported associations for FIB-4 are independent of the included cardiovascular and metabolic confounders, but not independently adjusted for chronological age as an external variable. All analyses were performed using SPSS 25.0 and R version 4.3.1, and the threshold for statistical significance was set at p < 0.05.

## 3 Results

### 3.1 Sample population inclusion process

The initial cohort comprised 121,409 adults (≥18 years) who completed a liver ultrasound examination. Subsequently, 15,544 individuals with complete datasets were included in the final analysis after the exclusion of participants with missing health information(Fig 1).

**Fig 1:**
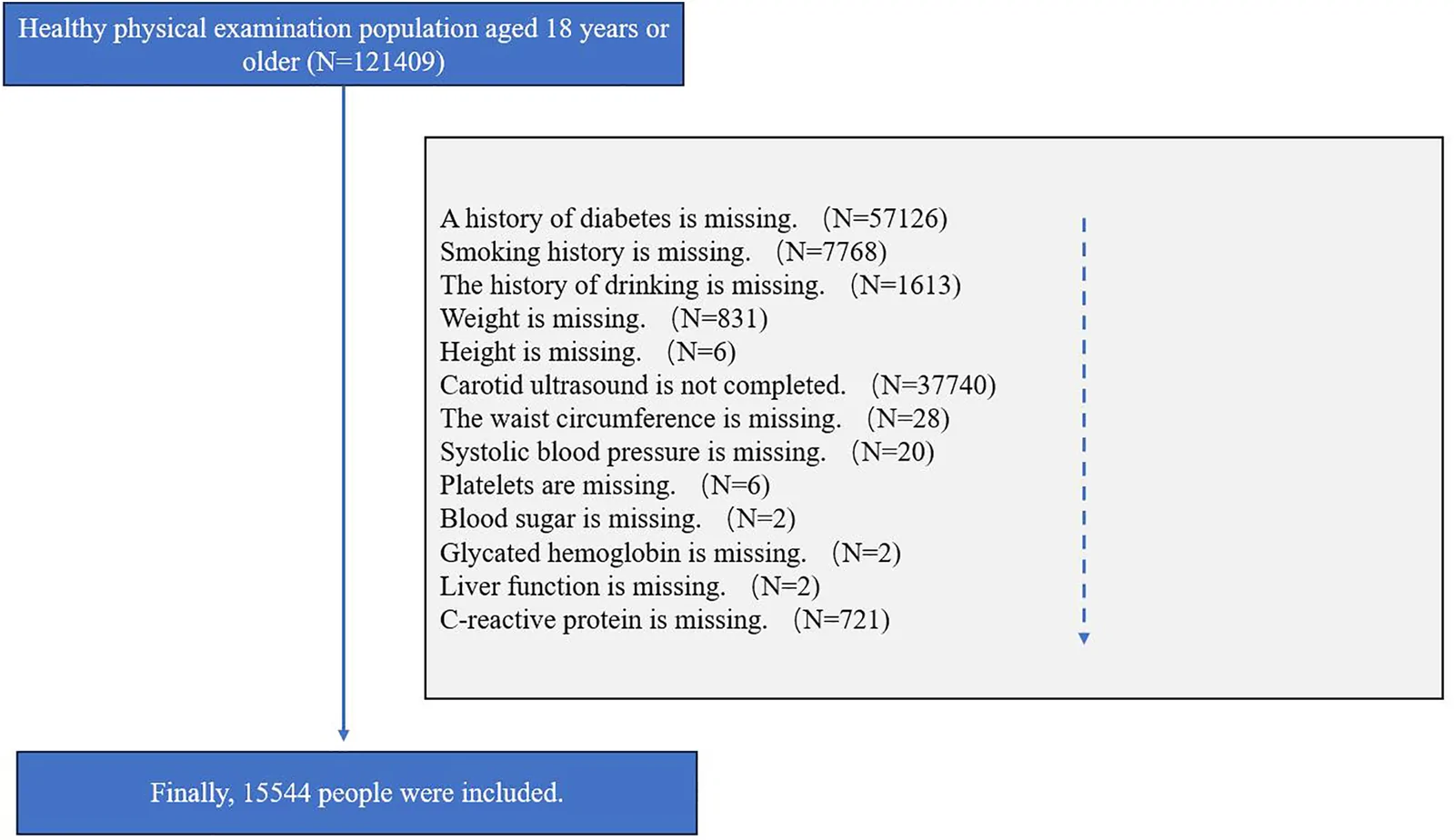
Sample population inclusion process.

### 3.2 Baseline data analysis of carotid plaque

Based on carotid ultrasound findings, the study population was categorized into two groups: a carotid plaque group (4839 patients) and a non‑carotid‑plaque control group (10705 patients). Comparative analysis(Table 1) revealed that the carotid plaque group was significantly older and had a higher proportion of males, smokers, and alcohol drinkers relative to the control group. Moreover, the prevalence of metabolic MAFLD was significantly greater in the carotid plaque group. Additionally, significant differences were observed in BMI, blood pressure, waist circumference, and various serum biochemical parameters between the two groups.

**Table 1:** Baseline data analysis of carotid plaque.

	Carotid plaque(n = 4839)	Non‑carotid‑plaque control(n=10705)	*p*
Age(years)	58(53,65)	50(43,56)	<0.001
Gender, n(%)	Men:3687(76.2%) Women:1152(23.8%)	Men:6891(64.4%) Women:3814(35.6%)	<0.001
Smoking, n(%)	Yes:1827(37.8%) No:3012(62.2%)	Yes:2935(27.4%) No:7770(72.6%)	<0.001
Drinking, n(%)	Yes:2346(48.5%) No:2493(51.5%)	Yes:4751(44.4%) No:5954(55.6%)	<0.001
MAFLD	Yes:3130(64.7%) No:1709(35.3%)	Yes:6010(56.1%) No:4695(43.9%)	<0.001
BMI (kg/m^2^)	24.88(23.07,26.95)	24.58(22.47,26.76)	<0.001
WC(cm)	88.00(82.00,94.00)	86.00(78.00,92.00)	<0.001
SBP(mmHg)	131(120,142)	124(113,135)	<0.001
DBP(mmHg)	76(68,83)	74(66,82)	<0.001
Platelet (10^9^/L)	204.00(173.00,238.00)	213.00(182.00,247.00)	<0.001
FBG(mmol/L)	5.38(4.93,6.11)	5.11(4.77,5.57)	<0.001
HbA1c(%)	5.80(5.50,6.20)	5.50(5.30,5.80)	<0.001
ALT(U/L)	22.00(16.00,30.00)	21.00(15.00,31.00)	<0.001
AST(U/L)	23.00(19.00,28.00)	22.00(18.00,26.00)	<0.001
ALP(U/L)	71.00(60.00,86.00)	68.00(56.00,82.00)	<0.001
GGT(U/L)	27.00(19.00,44.00)	25.00(16.00,42.00)	<0.001
TG(mmol/L)	1.49(1.06,2.14)	1.43(0.99,2.10)	<0.001
TC(mmol/L)	4.91(4.16,5.67)	4.99(4.38,5.65)	<0.001
HDL-c(mmol/L)	1.18(1.02,1.38)	1.21(1.04,1.42)	<0.001
LDL-c(mmol/L)	3.11(2.51,3.68)	3.15(2.68,3.63)	<0.001
UA(umol/L)	373.20(320.00,434.10)	364.50(301.85,428.00)	<0.001
25(OH)D(ng/mL)	20.42(16.05,25.23)	19.18(15.05,23.70)	<0.001
FIB-4	1.423(1.072,1.881)	1.098(0.840,1.446)	<0.001

Note: waist circumference(WC), Systolic blood pressure (SBP), diastolic blood pressure (DBP), fasting blood glucose(FBG), low-density lipoprotein (LDL-c), cholesterol (TC), triglycerides (TG), aspartate aminotransferase(AST), Alanine aminotransferase(ALT), glutamyl transpeptidase(GGT), alkaline phosphatase(ALP), glycosylated hemoglobin (HbA1c), uric acid (UA). *p* < 0.05 represents a statistical difference.

### 3.3 Association analysis of carotid plaque and MAFLD subtypes

To further investigate the associations between MAFLD subtypes and carotid plaque, three multivariable models were constructed(Table 2). In the unadjusted model, the subtype of MAFLD with T2DM exhibited the highest odds ratio (OR = 2.090), followed by lean MAFLD (OR = 1.917) and MAFLD with overweight/obesity (OR = 1.347). After adjustment for gender and age, lean MAFLD showed the numerically highest point estimate (OR = 1.469) among the three subtypes, relative to T2DM (OR = 1.428) and overweight/obesity (OR = 1.197); the differences across subtypes were not statistically significant. Further adjustment for smoking and alcohol consumption yielded consistent results, with lean MAFLD maintaining numerically highest risk (OR = 1.444), relative to the T2DM (OR = 1.354) and overweight/obesity (OR = 1.172) subgroups.

**Table 2:** Association analysis of carotid plaque and MAFLD subtypes.

	Crude (univariable)		Multivariable‑Model A (adjusted for age + gender)		Multivariable-Model B (adjusted for age, gender, smoking, alcohol consumption)	
Exposure	OR (95% CI)	*p*	OR (95% CI)	*p*	OR (95% CI)	p
**MAFLD**						
**Control**						
**Type 1**	2.090(1.780-2.456)	<0.001	1.428(1.194-1.709)	<0.001	1.354(1.130-1.622)	0.001
**Type 2**	1.347(1.253-1.449)	<0.001	1.197(1.099-1.304)	<0.001	1.172(1.076-1.277)	<0.001
**Type 3**	1.917(1.622-2.267)	<0.001	1.469(1.217-1.773)	<0.001	1.444(1.196-1.744)	<0.001

Note: Type 1: MAFLD with T2DM; Type 2: MAFLD with overweight/obesity; Type 3: Lean MAFLD. Crude (univariable): unadjusted regression model. Multivariable-Model A: adjusted for age and gender. Multivariable-Model B: further adjusted for smoking and alcohol consumption. *p* < 0.05 was considered statistically significant

### 3.4 Risk factor analysis of carotid plaque in MAFLD with T2DM group

To identify risk factors associated with carotid plaque, a cohort comprising 685 patients diagnosed with MAFLD with T2DM was selected. Univariate and multivariable logistic regression analyses were conducted(Table 3). The univariate analysis indicated a significant association between the FIB-4 index and carotid plaque (OR = 2.101).

**Table 3:** Risk factor analysis of carotid plaque in MAFLD with T2DM group.

	Univariate		Model 1		Model 2	
Exposure	OR (95% CI)	*p*	OR (95% CI)	*p*	OR (95% CI)	*p*
Age(years)	1.105(1.082-1.129)	<0.001	1.096(1.070-1.122)	<0.001	/	
Gender, n(%)	1.365(0.897-2.075)	0.146	/		/	
Smoking, n(%)	1.182(0.873-1.600)	0.280	/		/	
Drinking, n(%)	1.042(0.769-1.410)	0.729	/		/	
BMI (kg/m^2^)	0.929(0.881-0.981)	0.008	0.965(0.908-1.026)	0.253	0.930(0.877-0.986)	0.015
WC(cm)	0.990(0.973-1.009)	0.302	/		/	
SBP(mmHg)	1.020(1.009-1.031)	<0.001	1.013(1.001-1.025)	0.037	1.021(1.009-1.032)	0.001
DBP(mmHg)	0.990(0.975-1.005)	0.198	/		/	
Platelet (10^9^/L)	0.996(0.993-1.000)	0.025	0.999(0.995-1.002)	0.489	/	
FBG(mmol/L)	1.061(0.995-1.130)	0.069	0.968(0.868-1.079)	0.556	0.974(0.876-1.082)	0.618
HbA1c(%)	1.148(1.030-1.279)	0.013	1.153(0.956-1.390)	0.137	1.177(0.983-1.409)	0.076
ALT(U/L)	0.993(0.985-1.002)	0.126	/		/	
AST(U/L)	0.995(0.982-1.009)	0.501	/		/	
ALP(U/L)	0.998(0.990-1.005)	0.515	/		/	
GGT(U/L)	0.996(0.991-1.001)	0.087	0.998(0.993-1.003)	0.429	0.993(0.988-0.998)	0.006
TG(mmol/L)	0.961(0.898-1.029)	0.256	/		/	
TC(mmol/L)	1.133(0.994-1.293)	0.062	1.002(0.775-1.347)	0.877	0.923(0.707-1.205)	0.556
HDL-c(mmol/L)	1.419(0.801-2.515)	0.230	/		/	
LDL-c(mmol/L)	1.216(1.012-1.461)	0.037	1.229(0.846-1.784)	0.280	1.331(0.929-1.909)	0.119
UA(umol/L)	1.000(0.998-1.002)	0.894	/		/	
25(OH)D(ng/mL)	1.044(1.019-1.069)	<0.001	1.021(0.994-1.049)	0.128	1.037(1.011-1.064)	0.006
FIB-4	2.101(1.598-2.761)	<0.001	/		2.227(1.668-2.975)	<0.001

Note: *p* < 0.05 represents a statistical difference. Model 1: Candidate confounders selected via univariable screening (p < 0.10), without composite FIB‑4 index. Model 2: The same set of candidate confounders plus composite FIB‑4 index. Any of the four constituent parameters of FIB‑4 (age, platelet count, AST, ALT) selected in univariable screening were dropped from Model 2 to mitigate multicollinearity, given that all four variables contribute to FIB‑4 computation.

Given that FIB-4 is derived from parameters including age, platelet count, ALT and AST, two separate models were constructed to mitigate potential multicollinearity. Apart from the differential handling of FIB-4-related variables, the remaining adjusted covariates were identical between Model 1 and Model 2. Model 1 incorporated original clinical variables but excluded the composite FIB-4 index, whereas Model 2 included the composite FIB-4 index but excluded its constituent variables—specifically age, platelet count, AST, and ALT.

Multivariable analysis based on Model 1 identified age and systolic blood pressure(SBP) as significantly associated with carotid plaque. In Model 2 FIB-4 showed an association with carotid plaque (OR = 2.227).

### 3.5 Risk factor analysis of carotid plaque in MAFLD with overweight/obesity

A cohort of 7820 individuals with MAFLD and overweight/obesity was selected, and logistic regression analysis was performed(Table 4). Univariate analysis indicated that FIB-4 was significantly associated with carotid plaque (OR = 2.539).Multivariable analysis based on Model 1 showed that age, gender, smoking status, BMI, systolic blood pressure, platelet count, HbA1c and triglycerides were significantly associated with carotid plaque. In Model 2 (all pre-selected confounders adjusted, individual FIB-4 components excluded), FIB-4 was significantly correlated with carotid plaque risk (OR = 2.215).

**Table 4:** Risk factor analysis of carotid plaque in MAFLD with overweight/obesity.

	Univariate		Model 1		Model 2	
Exposure	OR (95% CI)	*p*	OR (95% CI)	*p*	OR (95% CI)	*p*
Age(years)	1.105(1.099-1.112)	<0.001	1.102(1.094-1.110)	<0.001	/	
Gender, n(%)	1.234(1.090-1.397)	0.001	1.337(1.124-1.591)	0.001	0.974 (0.825–1.149)	0.755
Smoking, n(%)	1.336(1.213-1.471)	<0.001	1.448(1.287-1.629)	<0.001	1.447 (1.292–1.621)	<0.001
Drinking, n(%)	1.042(0.948-1.146)	0.394	/		/	
BMI (kg/m^2^)	0.968(0.950-0.987)	0.001	0.946(0.914-0.979)	0.002	0.881 (0.853–0.910)	<0.001
WC(cm)	1.015(1.009-1.022)	<0.001	1.007(0.995-1.020)	0.236	1.035 (1.023–1.047)	<0.001
SBP(mmHg)	1.019(1.016-1.022)	<0.001	1.009(1.005-1.013)	<0.001	1.016 (1.013–1.020)	<0.001
DBP(mmHg)	0.997(0.993-1.002)	0.260	/		/	
Platelet (10^9^/L)	0.997(0.996-0.998)	<0.001	1.001(1.000-1.002)	0.038	/	
FBG(mmol/L)	1.224(1.178-1.273)	<0.001	0.949(0.884-1.019)	0.152	0.895 (0.836–0.959)	0.002
HbA1c(%)	1.574(1.478-1.676)	<0.001	1.366(1.219-1.529)	<0.001	1.683 (1.507–1.879)	<0.001
ALT(U/L)	0.995(0.993-0.998)	<0.001	1.001 (0.999–1.004)	0.262	/	
AST(U/L)	1.001(0.997-1.005)	0.594	/		/	
ALP(U/L)	1.000(0.998-1.003)	0.740	/		/	
GGT(U/L)	0.999(0.998-1.000)	0.079	1.000 (0.999–1.002)	0.532	0.999 (0.998–1.000)	0.029
TG(mmol/L)	0.914(0.883-0.945)	<0.001	0.933 (0.893–0.975)	0.001	0.901 (0.864–0.940)	<0.001
TC(mmol/L)	0.842(0.804-0.882)	<0.001	0.958 (0.821–1.118)	0.588	0.974 (0.840–1.130)	0.730
HDL-c(mmol/L)	1.024(0.845-1.241)	0.810	/		/	
LDL-c(mmol/L)	0.808(0.760-0.859)	<0.001	0.997 (0.817–1.218)	0.979	0.939 (0.775–1.137)	0.519
UA(umol/L)	0.999(0.999-1.000)	0.009	1.001 (1.000–1.001)	0.093	1.000 (0.999–1.001)	0.941
25(OH)D(ng/mL)	1.017(1.010-1.025)	<0.001	1.000 (0.992–1.008)	0.952	1.008 (1.001–1.016)	0.035
FIB-4	2.539(2.321-2.776)	<0.001	/		2.215 (2.015–2.433)	<0.001

Note: *p* < 0.05 represents a statistical difference. Model 1: Candidate confounders selected via univariable screening (p < 0.10), without composite FIB‑4 index. Model 2: The same set of candidate confounders plus composite FIB‑4 index. Any of the four constituent parameters of FIB‑4 (age, platelet count, AST, ALT) selected in univariable screening were dropped from Model 2 to mitigate multicollinearity, given that all four variables contribute to FIB‑4 computation.

### 3.6 Risk factor analysis of carotid plaque in lean MAFLD

Based on the predefined criteria, a total of 635 participants diagnosed with lean MAFLD were included in the analysis. Both univariate and multivariable logistic regression analyses were performed (Table 5). The univariate analysis indicated that the FIB‑4 index was significantly associated with an increased risk of carotid plaque (OR = 1.836).

**Table 5:** Risk factor analysis of carotid plaque in Lean MAFLD.

	Univariate		Model 1		Model 2	
Exposure	OR (95% CI)	*p*	OR (95% CI)	*p*	OR (95% CI)	*p*
Age(years)	1.101(1.078-1.124)	<0.001	1.111 (1.081–1.141)	<0.001	/	
Gender, n(%)	1.704(1.231-2.359)	0.001	0.779 (0.450–1.349)	0.373	0.844 (0.507–1.406)	0.516
Smoking, n(%)	2.244(1.567-3.214)	<0.001	2.336 (1.412–3.864)	0.001	2.186 (1.370–3.490)	0.001
Drinking, n(%)	1.693(1.221-2.348)	0.002	1.518 (0.956–2.412)	0.077	1.304 (0.847–2.009)	0.228
BMI (kg/m^2^)	0.967(0.813-1.151)	0.706	/		/	
WC(cm)	1.061(1.032-1.091)	<0.001	1.038 (0.999–1.078)	0.055	1.047 (1.010–1.085)	0.013
SBP(mmHg)	1.031(1.020-1.041)	<0.001	1.006 (0.989–1.023)	0.526	1.035 (1.019–1.050)	<0.001
DBP(mmHg)	1.020(1.004-1.036)	0.013	1.019 (0.992–1.046)	0.170	0.984 (0.961–1.007)	0.171
Platelet (10^9^/L)	0.998(0.994-1.001)	0.160	/		/	
FBG(mmol/L)	1.223(1.070-1.399)	0.003	0.952 (0.745–1.217)	0.696	0.969 (0.765–1.227)	0.795
HbA1c(%)	1.326(1.096-1.603)	0.004	1.126 (0.794–1.598)	0.504	1.257 (0.902–1.750)	0.177
ALT(U/L)	0.996(0.988-1.005)	0.406	/		/	
AST(U/L)	1.005(0.994-1.016)	0.380	/		/	
ALP(U/L)	1.006(0.999-1.012)	0.071	0.998 (0.990–1.006)	0.665	1.001 (0.993–1.008)	0.874
GGT(U/L)	1.004(1.001-1.006)	0.013	1.003 (1.000–1.007)	0.052	1.001 (0.998–1.004)	0.346
TG(mmol/L)	0.966(0.875-1.065)	0.484	/		/	
TC(mmol/L)	0.843(0.730-0.973)	0.020	0.811 (0.502–1.310)	0.391	0.846 (0.537–1.333)	0.472
HDL-c(mmol/L)	1.911(1.054-3.463)	0.033	1.644 (0.734–3.681)	0.227	2.386 (1.106–5.145)	0.027
LDL-c(mmol/L)	0.731(0.599-0.892)	0.002	0.959 (0.516–1.785)	0.896	0.875 (0.486–1.578)	0.658
UA(umol/L)	1.001(0.999-1.003)	0.441	/		/	
25(OH)D(ng/mL)	1.000(0.978-1.022)	0.972	/		/	
FIB-4	1.836(1.388-2.429)	<0.001	/		1.401 (1.044–1.881)	0.025

Note: *p* < 0.05 represents a statistical difference. Model 1: Candidate confounders selected via univariable screening (p < 0.10), without composite FIB‑4 index. Model 2: The same set of candidate confounders plus composite FIB‑4 index. Any of the four constituent parameters of FIB‑4 (age, platelet count, AST, ALT) selected in univariable screening were dropped from Model 2 to mitigate multicollinearity, given that all four variables contribute to FIB‑4 computation.

Analysis based on Model 1 identified age, smoking status as significant factors associated with carotid plaque. In Model 2 (all pre‑selected confounders adjusted, individual FIB‑4 components excluded), the FIB‑4 index was significantly associated with carotid plaque risk (OR = 1.401).

## 4 Discussion

MASLD, as the successor to MAFLD, encompasses a broader patient population. However, since our research aims to investigate the role of strict metabolic dysfunction, the MAFLD criteria remain the more appropriate and precise choice for delineating our study cohort. Our study revealed a noteworthy shift in the association between MAFLD subtypes and carotid plaque upon adjustment for confounders. While the diabetes-related MAFLD subtype exhibited the highest odds ratio (OR) in the unadjusted model, lean MAFLD emerged as the subtype with a notably strong independent association with carotid plaque after adjusting for gender, age, smoking, and alcohol consumption. Given that the FIB-4 score is a composite metric derived from age, platelet count, AST, and ALT, its inclusion in models containing these individual components may result in potential multicollinearity, thereby obscuring its independent association. To address this, Model 2 was constructed to evaluate the independent association of the composite FIB-4. This model retained all cardiovascular and metabolic confounders while any FIB‑4 components selected via univariable screening were removed to mitigate multicollinearity. Model 2 reflects real-world usage and confirms that FIB‑4 retains a significant association with carotid plaque risk when considered separately from its constituent components, FIB-4 retains a significant association with carotid plaque risk. This supports the use of FIB-4 as a simple and integrated non-invasive tool for initial risk stratification in clinical settings.

Carotid atherosclerosis is considered the leading cause of ischemic stroke [11]. Carotid plaque can be identified via ultrasound, which is a recognized indicator of subclinical atherosclerosis, and the evaluation of carotid plaque can help improve the prediction of cardiovascular risk [12]. Therefore, strategies for the early detection and intervention of carotid plaque can significantly improve cardiovascular risk prognosis. The adjusted analysis showed that the risk of carotid plaque in lean MAFLD remains independent of traditional cardiovascular risk factors, suggesting an intrinsic disease‑related risk. While unadjusted models showed a high risk of MAFLD with T2DM, this is likely confounded by factors like age and sex. After adjustment, its observed point estimate appeared lower than that of lean MAFLD, which retained a strong independent association, although the confidence intervals of the three subgroups substantially overlapped, providing no clear evidence of a difference.

An association was observed between MAFLD/MASLD and cardiovascular disease risk, with the influence of metabolic health or alcohol consumption outweighing that of obesity [13]. Studies have found that Lean MAFLD often exhibits different risk factors due to its association with metabolic dysregulation compared to MAFLD with overweight/obesity, and it has been confirmed that increased visceral fat, muscle loss, and increased cardiometabolic and renal comorbidities are associated with Lean MAFLD [14]. Studies have suggested that lean MAFLD had a more significant mortality rate than non-lean MAFLD, and the mechanism is related to increased ROS levels, decreased GDF-15, induced telomere shortening and reduced telomere activity in hepatocytes [15]. A study indicated that, irrespective of metabolic irregularities or other concurrent illnesses, individuals with lean MAFLD or MAFLD with T2DM face a greater risk of cardiovascular disease compared to those with MAFLD who are overweight or obese [16]. Our findings carry significant clinical implications. This suggests that the atherogenic risk in Lean MAFLD is not driven by these conventional factors but rather by intrinsic pathophysiological mechanisms, potentially involving genetic factors, visceral adiposity, or a distinct metabolic phenotype. Consequently, our results argue against the perception of Lean MAFLD as a benign condition and call for vigilant cardiovascular risk assessment and management in this specific patient population.

Although the prediction model of the original indicator showed optimal discriminant ability, multivariable logistic regression analysis revealed that the FIB-4 index showed an association with carotid plaque independent of measured cardiovascular-metabolic confounders (chronological age is embedded within the FIB‑4 calculation and not adjusted as a separate covariate), which supports our choice to include the FIB-4 index in the final model to better explain its clinical value. The Fibrosis 4 score (FIB-4) is based on routine clinical and laboratory variables and has the advantages of broad applicability, accessibility, and safety, and can be used as a non-invasive measurement tool for liver fibrosis [17]. In this study, the FIB-4 index was found to be significantly associated with carotid plaque risk in all three MAFLD subtypes. This strongly suggests that elevated liver‑fibrosis risk, as reflected by the FIB‑4 index, may represent a shared key pathophysiological pathway linking hepatic disorders to vascular damage. Liver fibrosis serves as a critical link between hepatic pathology and extrahepatic vascular diseases, and research indicates that it may directly contribute to vascular endothelial dysfunction, increase arterial stiffness, and exacerbate systemic inflammation [18]. Accumulating evidence demonstrates a clear association between liver pathology outcomes and cardiovascular disease risk, with cirrhosis constituting the most significant risk factor for cardiovascular events, followed by fibrotic steatohepatitis, non-fibrotic steatohepatitis, and simple steatosis under MASLD classification—all independent of age and other cardiometabolic risk factors [19]. MAFLD, now widely recognized as MASLD, remains a highly prevalent yet frequently underdiagnosed condition, with the FIB-4 score identified as a marker linked to elevated risks of end-stage liver disease among young, otherwise healthy adults [20]. Recent research has indicated that scores such as the aspartate aminotransaminase-to-platelet ratio index (APRI), the fibrosis-4 (FIB-4) index, the NAFLD score (NFS), and the Hepamet fibrosis score (HFS) are viable tools for evaluating liver fibrosis in individuals with MASLD, among which the FIB-4 and APRI scores have demonstrated higher diagnostic accuracy [21]. In conclusion, the FIB-4 score not only consistently reveals a strong association between liver fibrosis and vascular lesions across various subtypes of MAFLD but also exhibits high clinical applicability and diagnostic significance, thereby supporting its role as a core tool for assessing liver fibrosis and related cardiovascular risks across diverse populations.

Research has indicated that liver fibrosis acts as a surrogate marker for both all-cause and liver-specific mortality in individuals with MAFLD, with particular emphasis on the greater clinical importance of assessing liver fibrosis compared to evaluating the severity of fat accumulation in such individuals [22]. Studies have indicated that insulin resistance and inflammation participate in the pathophysiological processes of liver fibrosis [23]. Consequently, the core mechanisms underlying liver fibrosis might also play a role in the development of carotid plaques. Meanwhile, despite having a normal body weight, individuals with lean MAFLD frequently exhibit more “hidden obesity,” such as increased visceral fat accumulation-or possess genetic susceptibility. These characteristics may render them more vulnerable to developing significant vascular inflammation or liver fibrosis. Our findings are consistent with previous studies reporting that the FIB-4 index is associated with an increased risk of vascular lesions [24]. However, our work extends prior observations by showing that FIB‑4 is associated with carotid plaque across all MAFLD subgroups, after accounting for available cardiovascular and metabolic confounders. These findings add subgroup‑specific evidence to inform clinical risk‑stratification.The findings of this study indicate that the FIB-4 index ought to be routinely computed for all individuals diagnosed with MAFLD, irrespective of their BMI. Notably, for individuals with lean MAFLD, even in the absence of conventional cardiovascular risk factors, they should be classified as high-risk individuals and thus undergo more proactive carotid ultrasound screening as well as targeted cardiovascular risk intervention measures. This study has several limitations that should be acknowledged. First, as a cross-sectional study, the design does not allow for the establishment of causal relationships between FIB-4 and atherosclerosis. Second, the absence of insulin testing and incomplete data on alcohol consumption may have introduced diagnostic omissions. Furthermore, although multivariable adjustment was performed, the age component incorporated in the FIB-4 index may be partially collinear with other age-related risk factors for atherosclerosis. In addition, partial electronic medical records lack quantitative daily alcohol intake data; only patients with clear heavy drinking records were excluded, which may introduce minor residual confounding effects. Future prospective research with standardized alcohol recording is needed to further verify our results. Only fasting blood glucose and glycated hemoglobin data were available in our physical examination database, and post-load glycemic indicators including OGTT 2-hour glucose were not routinely collected. Thus, some participants with isolated impaired glucose tolerance (prediabetes) could not be identified, which may underestimate the prevalence of metabolic dysfunction in the study population. This incomplete glycemic evaluation constitutes a limitation of the present retrospective cohort analysis. The BMI threshold of 23 kg/m^2^ used to define lean MAFLD is only applicable to Asian populations, and this single ethnic population with exclusive Asian BMI cut-offs may limit the generalizability of our findings to other racial groups. Hepatic steatosis was screened via abdominal ultrasound rather than transient elastography, which exhibits higher sensitivity and specificity for fatty liver quantification; this imaging modality difference constitutes a study limitation.Therefore, large-scale prospective cohort studies are warranted in the future to validate these findings.

## 5 Conclusions

In summary, the present study demonstrates that lean MAFLD constitutes a potent independent risk factor for carotid plaque, with its point estimate numerically higher than that of diabetes‑related MAFLD, although confidence intervals overlapped across subgroups. Furthermore, our findings indicate that the readily available FIB-4 index acts as a valuable marker associated with elevated carotid‑plaque risk. These results underscore the need for clinicians to recognize the significant cardiovascular implications of lean MAFLD and support the integration of FIB-4 into standardized cardiovascular risk stratification protocols to facilitate early intervention and improve individual prognosis.
